# Comparing therapeutic effects of hematopoietic stem cell transplantation, tyrosine kinase inhibitors and chemotherapy in adult patients with Philadelphia chromosome-positive acute lymphoblastic leukemia: a systematic review and meta-analysis

**DOI:** 10.3389/fonc.2025.1627825

**Published:** 2025-10-15

**Authors:** Xiaohui Gao, Hui Zeng, Fei Sun, Xiaoyan Zhao, Haibing Wu, Minchao Yan, Yuan Li, Qinyan Fu, Gang Zhang

**Affiliations:** ^1^ Departments of Pediatrics, The Affiliated Hospital of Jiaxing University, Jiaxing, Zhejiang, China; ^2^ Departments of Hematology, The Affiliated Hospital of Jiaxing University, Jiaxing, Zhejiang, China

**Keywords:** Philadelphia chromosome-positive, acute lymphoblastic leukemia, tyrosine kinase inhibitor, hematopoietic stem cell transplantation, meta-analysis

## Abstract

**Objective:**

Both hematopoietic stem cell transplantation (HSCT) and chemotherapy combined with tyrosine kinase inhibitors (TKIs) have shown therapeutic efficacy in patients with Philadelphia chromosome-positive (Ph+) acute lymphoblastic leukemia (ALL). This study aimed to compare the clinical outcomes of HSCT and TKI-combined chemotherapy regimens in Ph+ ALL through a meta-analysis.

**Methods:**

We systematically searched PubMed (from 1966), Embase (from 1974), and the Cochrane Library (from 1993) up to April 30, 2025, for eligible studies. Overall survival (OS) and disease-free survival (DFS) were evaluated using hazard ratios (HRs) with 95% confidence intervals (CIs), while relapse risk was assessed using odds ratios (ORs) with 95%CIs. A random-effects model was applied for all analyses.

**Results:**

The meta-analysis included 35 studies involving 3,827 patients with Ph+ ALL. Allogeneic HSCT (allo-HSCT) was associated with significantly better OS (HR: 0.60; 95% CI: 0.45–0.81; *P* = 0.001) and DFS (HR: 0.40; 95% CI: 0.30–0.54; *P* < 0.001) compared to TKI-based chemotherapy. No significant differences in OS (HR: 0.97; 95% CI: 0.70–1.34; *P* = 0.845) or DFS (HR: 0.92; 95% CI: 0.67–1.26; *P* = 0.605) were observed between allo-HSCT and autologous HSCT (auto-HSCT). Moreover, allo-HSCT was associated with a significantly lower relapse risk than both TKI-based chemotherapy (OR: 0.28; 95% CI: 0.16–0.51; *P* < 0.001) and auto-HSCT (OR: 0.39; 95% CI: 0.27–0.54; *P* < 0.001).

**Conclusion:**

This meta-analysis demonstrates that allo-HSCT provides superior survival outcomes compared to TKI-based chemotherapy in patients with Ph+ ALL. Although survival outcomes are similar between allo-HSCT and auto-HSCT, allo-HSCT is associated with a significantly reduced risk of relapse.

**Systematic Review Registration:**

https://www.crd.york.ac.uk/prospero/, identifier INPLASY202550012.

## 1 Introduction

Philadelphia chromosome-positive acute lymphoblastic leukemia (Ph+ ALL) is a distinct subtype of ALL characterized by the presence of the BCR::ABL1 fusion gene, which encodes a constitutively active BCR-ABL1 tyrosine kinase oncoprotein (1). In adults with ALL, Ph+ ALL accounts for 20%–25% of cases, whereas its incidence in pediatric patients ranges from 3%–5% (2, 3). This genetic aberration serves not only as a critical diagnostic marker but also informs risk stratification and guides targeted treatment strategies.

Before the introduction tyrosine kinase inhibitors (TKIs), the standard management of Ph+ ALL relied on intensive chemotherapy, with overall survival (OS) rates remaining below 40% (4). The integration of TKIs into chemotherapy regimens has significantly improved clinical outcomes, achieving complete hematologic remission in 94%–100% of patients and reducing induction-related mortality to less than 5% (5, 6). As a result, TKI-based chemotherapy has become the first-line treatment for newly diagnosed Ph+ ALL, leading to substantial improvements in both remission rates and long-term survival (7).

Allogeneic hematopoietic stem cell transplantation (allo-HSCT) continues to be the standard consolidation therapy for eligible patients with suitable donors, supported by robust evidence of its efficacy (8–10). Both HLA-matched related and unrelated donor transplants have demonstrated favorable outcomes. A multicenter study conducted in Southwest China further indicated that haploidentical HSCT (haplo-HSCT) offers survival benefits comparable to those of matched sibling transplantation in Ph+ ALL patients (11).

In the TKI era, autologous HSCT (auto-HSCT) has emerged as a context-dependent alternative, particularly for patients without access to an allo-HSCT donor or those deemed medically unsuitable for allo-HSCT. TKIs can effectively reduce tumor burden to achieve minimal residual disease (MRD)-negative or low-MRD status prior to auto-HSCT, thereby mitigating key limitations of this approach—such as the absence of a graft-versus-leukemia effect and the risk of graft contamination. In TKI-pretreated patients, relapse due to graft contamination is now reported in less than 5% of cases (12). Importantly, data from the European Society for Blood and Marrow Transplantation Acute Leukemia Working Group indicate that myeloablative auto-HSCT can provide leukemia-free survival comparable to that of allo-HSCT in Ph+ ALL patients who maintain complete molecular remission (CMR) for more than three months following TKI therapy (13).

Despite these therapeutic advances, the optimal treatment strategy for Ph+ ALL remains a subject of debate. To address this uncertainty, we conducted a systematic review and meta-analysis to compare the therapeutic efficacy of HSCT and TKI-combined chemotherapy regimens in patients with Ph+ ALL.

## 2 Methods

### 2.1 Search strategy and selection criteria

This systematic review was conducted in accordance with the Preferred Reporting Items for Systematic Reviews and Meta-Analyses guidelines (14). The study protocol was registered on the INPLASY platform (No: INPLASY202550012). Eligible studies were those that directly compared clinical outcomes between HSCT and TKI-based chemotherapy in patients with Ph+ ALL. No restrictions were placed on publication language. We searched PubMed (from 1966), Embase (from 1974), and the Cochrane Library (from 1993) from their earliest available dates up to April 30, 2025. The search strategy incorporated Boolean operators and the Medical Subject Heading (MeSH) term: “Philadelphia chromosome-positive acute lymphoblastic leukemia”. Additional searches were performed in ClinicalTrials.gov, the World Health Organization International Clinical Trials Registry Platform (WHO ICTRP), conference proceedings from major hematology meetings, and reference lists of included studies and relevant systematic reviews published in the past five years. Studies were excluded if they involved upfront immunotherapy (e.g., blinatumomab, inotuzumab ozogamicin) as part of the initial induction or consolidation therapy in either the intervention or control group. This criterion was applied to ensure a consistent comparison between the core therapeutic strategies of interest—HSCT and TKI-based chemotherapy. Studies in which immunotherapy was used solely as salvage treatment for relapsed disease were retained.

To minimize selection bias, two reviewers independently screened studies in a blinded manner. Initial screening was based on titles and abstracts to exclude clearly irrelevant studies. Subsequently, full-text articles were reviewed to determine final eligibility. Any disagreements were resolved through discussion or by consultation with a third senior reviewer. The inclusion criteria were structured using the PICOS framework: (1) Population: patients with Ph+ ALL confirmed by cytogenetics or molecular biology; (2) Intervention: HSCT (allogeneic or autologous); (3) Comparison: TKI combined with intensive chemotherapy; (4) Outcomes: OS, disease-free survival (DFS), and relapse incidence; and (5) Study design: prospective or retrospective comparative studies.

### 2.2 Data collection and quality assessment

Data extraction covered the following information: first author, publication year, study design, country, sample size, mean age, type of TKI, disease status, details of the intervention and control groups, and reported outcomes. Methodological quality was assessed using the Newcastle-Ottawa Scale (NOS) for observational studies (15). The NOS assigns a maximum of 9 points across three domains: (1) Selection (4 points): representativeness of the exposed cohort, selection of the non-exposed cohort, ascertainment of exposure, and demonstration that the outcome was not present at baseline; (2) Comparability (2 points): control for confounding factors; and (3) Outcome (3 points): assessment of outcome, adequacy of follow-up, and completeness of follow-up. Studies scoring ≥7, 5–6, and <5 were considered high, moderate, and low quality, respectively. A three-step quality control process was implemented: (1) Two researchers independently performed quality assessments using a standardized electronic form; (2) Discrepancies of ≥ 2 points were resolved by a senior methodologist according to the NOS manual; and (3) All extracted data were double-entered and cross-verified. Logical inconsistencies were corrected by referring to the original source documents. A final check for missing data was conducted before database lock.

### 2.3 Statistical analysis

Treatment effects for OS and DFS were summarized as hazard ratios (HRs) with 95% confidence intervals (CIs). Relapse incidence was analyzed using odds ratios (ORs) with 95%CIs. All meta-analyses were performed using the DerSimonian–Laird random-effects model to incorporate potential clinical heterogeneity (16, 17). Heterogeneity was assessed using Cochran’s Q test (significance threshold: *P* < 0.10) and the *I*² statistic, with *I^2^* ≥ 50% indicating substantial heterogeneity (18, 19). Sensitivity analyses were conducted using the leave-one-out method, supported by Baujat plots to identify influential studies (20). Prespecified subgroup analyses were performed based on study design, country, intervention type, TKI type, and study quality. Between-subgroup differences were evaluated using mixed-effects meta-regression with restricted maximum likelihood estimation. Permutation tests were applied to avoid normality assumptions (21). Publication bias was evaluated using contour-enhanced funnel plots, Egger’s regression test, and Begg’s rank correlation test (22, 23). If significant asymmetry was detected (*P* < 0.10), the trim-and-fill method was used to estimate adjusted effect sizes (24). All statistical tests were two-sided, with a significance level of α = 0.05. Analyses were performed using STATA version 12.0 (StataCorp, College Station, TX, USA) with the “*metan*” package for meta-analysis.

## 3 Results

### 3.1 Literature search

**Figure 1:**
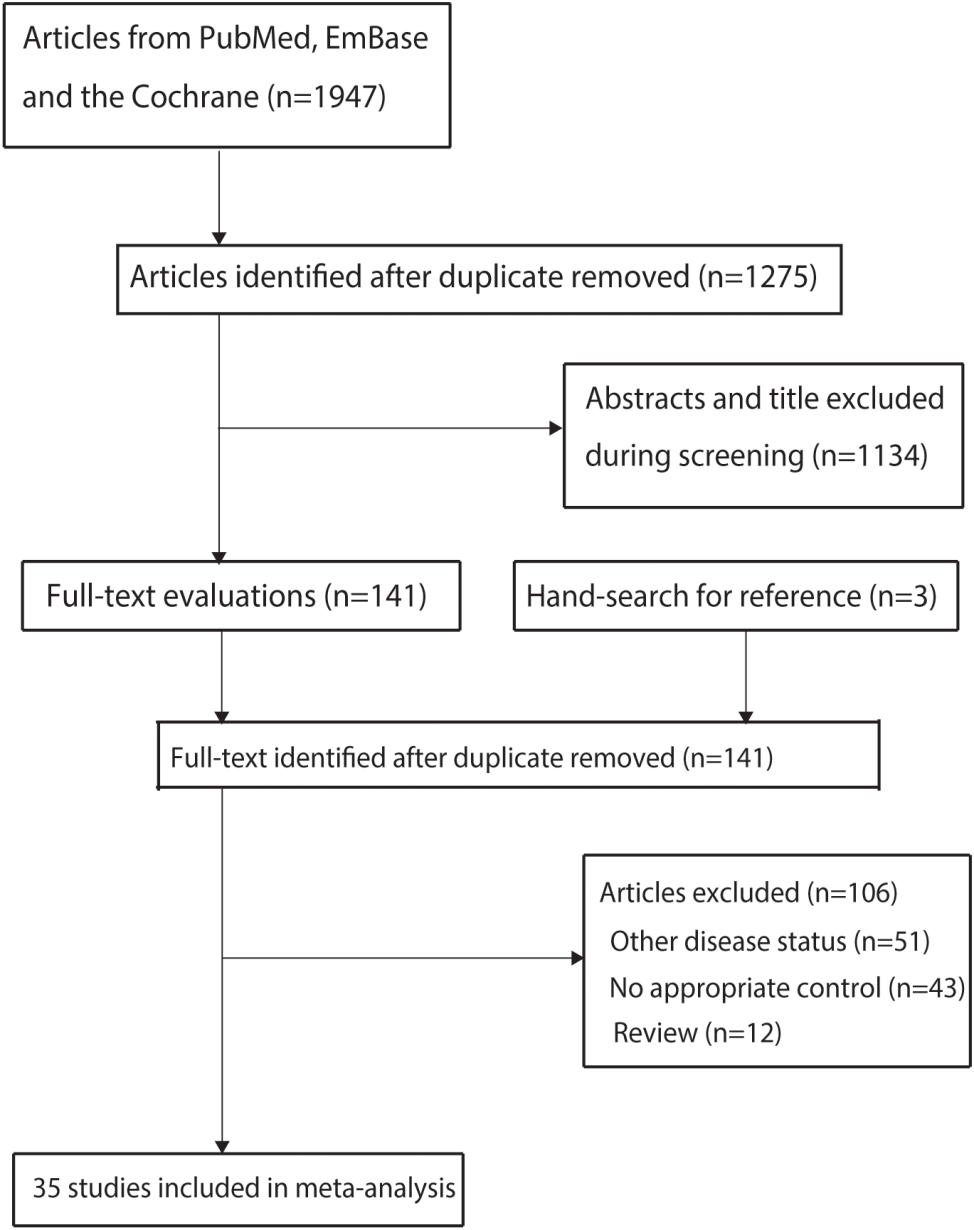
PRISMA flow diagram illustrating the literature search and study selection process.

A total of 1,947 articles were initially identified through electronic database searches. After removing duplicates, 1,275 articles remained. Screening of titles and abstracts led to the exclusion of 1,134 articles. The remaining 141 studies underwent full-text review, of which 35 met the inclusion criteria and were included in the meta-analysis (13, 25–58). Manual searches of the reference lists of these studies identified three additional potentially eligible articles; however, further verification confirmed that these had already been captured in the electronic search and were therefore excluded. The study selection process is summarized in **Figure 1**.

### 3.2 Study characteristics

The baseline characteristics of the included studies and patients are presented in **Table 1**. The 35 eligible studies, published between 2010 and 2024, involved a total of 3,827 patients with Ph+ ALL, with individual study sample sizes ranging from 18 to 569. Eleven studies were prospective in design, while 24 were retrospective. Geographically, 16 studies were conducted in Western countries and 19 in Eastern countries. Among the chemotherapy arms of the included studies, the initial TKI agents used were distributed as follows: imatinib (first-generation) in 17 studies, dasatinib (second-generation) in 3 studies, nilotinib (second-generation) in 2 studies, ponatinib (third-generation) in 2 studies, and mixed TKI cohorts in 10 studies. One study did not specify the TKI type used. Quality assessment using the NOS yielded the following scores: 10 studies scored 9 points, 7 scored 8 points, 13 scored 7 points, 2 scored 6 points, and 3 scored 5 points.

**Table 1:** Baseline characteristics of identified studies and involved patients.

Study	Study design	Country	Sample size	Median age (years)	TKI type	Disease status	Intervention and control	Study quality
Bassan 2010 (25)	Prospective	Italy	54	47.1	Imatinib	BCR-ABL1 P190 (69.0%); BCR-ABL1 P210 (28.0%); t (9:22): 70.2%; additional aberrations: 33.0%; CD10+: 94.0%; CD20+: 37.0%	HSCT (Allo-HSCT; auto-HSCT); NILG protocol 09/00	9
Li 2010 (26)	Retrospective	China	63	34.6	Imatinib	BCR-ABL1 P190 (61.9%); BCR-ABL1 P210 (19.0%)	Allo-HSCT; Vincristine, daunorubicin, cyclophosphamide, prednisone and L-asparagine	6
Thyagu 2012 (27)	Retrospective	Canada	28	46.0	Imatinib	CD10+: 96.4%; CD20+: 53.6%	Allo-HSCT; Doxorubicin, vincristine, asparaginase, dexamethasone plus mercaptopurine plus methotrexate	7
Pfeifer 2012 (28)	Retrospective	Germany	115	NA	Imatinib	NA	Allo-HSCT; standard chemotherapy regimen	5
Tanguy-Schmidt 2013 (29)	Prospective	France	43	43.0	Imatinib	NA	HSCT (Allo-HSCT; auto-HSCT); Etoposide, HD-ara, and mitoxantrone	7
Konopacki 2013 (30)	Retrospective	France	18	53.6	Imatinib or dasatinib	NA	Allo-HSCT; Hyper-CVAD or GRAALL protocol	7
Wetzler 2014 (31)	Prospective	USA	34	45.0	Imatinib	t (9:22): 11.8%; t (9:22)+additional aberrations: 44.1%	Allo-HSCT; Auto-HSCT	7
Fielding 2014 (32)	Prospective	UK	130	NA	Imatinib	NA	HSCT (Allo-HSCT; auto-HSCT); standard chemotherapy regimen	9
Chalandon 2015 (33)	Prospective	France	196	NA	Imatinib	t (9:22): 92.2%	Allo-HSCT; Auto-HSCT	9
Ravandi 2015 (34)	Prospective	USA	72	55.0	Dasatinib	BCR-ABL ela2: 72.2%; b2a2: 18.1%; b2a2+b3a2: 2.8%; b3a2/e1a3: 5.6%	Allo-HSCT; Hyper-CVAD	9
Daver 2015 (35)	Prospective	USA	39	51.0	Imatinib	BCR-ABL1 P190 (67.0%); BCR-ABL1 P210 (33.0%)	Allo-HSCT; Hyper-CVAD	7
Sun 2015 (36)	Retrospective	China	62	NA	Imatinib	NA	Allo-HSCT; vincristine, daunorubicin, cyclophosphamide and prednisone	8
Togasaki 2015 (37)	Retrospective	Japan	22	53.0	Imatinib	NA	Allo-HSCT; Hyper-CVAD or Ph-positive ALL 202 protocol	7
Kim 2015 (38)	Prospective	Korea	82	47.0	Nilotinib	BCR-ABL1 transcript, major: 28.0%; minor: 65.0%	Allo-HSCT; daunorubicin, vincristine, prednisone, HD-Ara, and etoposide	9
Tan 2015 (39)	Prospective	China	36	NA	Imatinib	NA	Allo-HSCT; Auto-HSCT	5
Kuang 2016 (40)	Retrospective	China	49	NA	Imatinib	BCR-ABL1 P190 (70.6%); BCR-ABL1 P210 (25.5%)	Allo-HSCT; vincristing and dexamethasone	9
Ravandi 2016 (41)	Prospective	USA	78	44.0	Dasatinib	NA	Allo-HSCT; Hyper-CVAD	8
Kanfar 2016 (42)	Retrospective	Saudi Arabia	133	NA	Imatinib	NA	Allo-HSCT; DFCI pediatric ALL protocol	7
Kozlowski 2017 (43)	Retrospective	Sweden	42	64.8	Imatinib or dasatinib	BCR-ABL: 35.0%	Allo-HSCT; EWALL-backbone therapy, ABCDV protocol, hyper-CVAD, or daunorubicin/cytarabine	7
Fujisawa 2017 (44)	Retrospective	Japan	65	47.8	Imatinib	t (9:22): 35.3%; additional aberrations: 64.7%; CD20+: 27.9%; CD13+: 52.9%; CD33+: 35.3%; CD34+: 94.1%; BCR-ABL1 transcript, major: 25.0%; minor: 70.6%	Allo-HSCT; daunorubicin, cyclophosphamide, vincristine, and prednisolone	9
Liu 2017 (45)	Retrospective	China	86	NA	Imatinib, nilotinib or dasatinib	NA	Allo-HSCT; Auto-HSCT	5
Hatta 2018 (46)	Retrospective	Japan	96	NA	Imatinib	t (9:22): 51.0%; additional aberrations: 49.0%; CD13/CD33+: 43.0%	Allo-HSCT; Ph-positive ALL 202 protocol	9
Jabbour 2018 (47)	Prospective	USA	62	NA	Ponatinib	BCR-ABL1 P190 (74.0%); BCR-ABL1 P210 (25.0%)	Allo-HSCT; Hyper-CVAD	9
Giebel 2018 (13)	Retrospective	Europe	569	40.7	NA	NA	Allo-HSCT; Auto-HSCT	8
Wang 2018 (48)	Retrospective	China	133	37.0	Imatinib	BCR-ABL1 P190 (71.4%); BCR-ABL1 P210 (27.1%); t (9:22): 40.6%	Allo-HSCT; Hyper-CVAD	7
Agrawal 2019 (49)	Retrospective	India	41	35.0	Imatinib or dasatinib	NA	Allo-HSCT; Hyper-CVAD	7
Chang 2019 (50)	Retrospective	China	70	45.0	Dasatinib	BCR-ABL1 P190 (65.7%); BCR-ABL1 P210 (34.3%)	Allo-HSCT; Hyper-CVAD, BFM-like, or pediatric-inspired ALL regimen	8
Liu 2019 (51)	Retrospective	China	27	40.0	Nilotinib	BCR-ABL1 P190 (80.0%); BCR-ABL1 P210 (20.0%)	Allo-HSCT; cyclophosphamide, vincristine, cytarabine, teniposide, dexamethasone	7
Wang 2020 (52)	Retrospective	China	134	38.5	Imatinib or dasatinib	BCR-ABL ela2: 73.1%; e13a2 or e14a2: 26.9%	Allo-HSCT; Hyper-CVAD, or daunorubicin, vincristine, and prednisolone	8
Ghobadi 2020 (53)	Retrospective	USA	186	52.1	Imatinib, dasatinib, or ponatinib	NA	Allo-HSCT; Hyper-CVAD	9
Lyu 2021 (54)	Retrospective	China	119	NA	Imatinib, dasatinib, or nilotinib	BCR-ABL1 P190 (73.1%); BCR-ABL1 P210 (22.7%); t (9:22): 52.9%; additional aberrations: 29.4%	Allo-HSCT; Auto-HSCT	7
Wu 2022 (55)	Retrospective	China	198	38.9	Imatinib, dasatinib, or nilotinib	BCR-ABL1 P190 (39.9%); BCR-ABL1 P210 (24.7%)	Allo-HSCT; Hyper-CVAD or CALLG2008	8
Othman 2022 (56)	Retrospective	USA	22	53.4	Ponatinib	CD20+: 50.0%; BCR-ABL1 P190 (27.2%); BCR-ABL1 P210 (13.6%)	Allo-HSCT; Hyper-CVAD	6
Badar 2024 (57)	Retrospective	USA	431	52.0	Imatinib, dasatinib, nilotinib, or ponatinib	BCR-ABL1 P190 (75.0%); BCR-ABL1 P210 (25.0%)	Allo-HSCT; intensive or non-intensive chemotherapy	7
Hu 2024 (58)	Retrospective	China	292	38.0	Imatinib or dasatinib	BCR-ABL1 P190 (70.0%); BCR-ABL1 P210 (30.0%)	Allo-HSCT; Hyper-CVAD	8

NA indicated “not available.”

### 3.3 Overall survival

**Figure 2:**
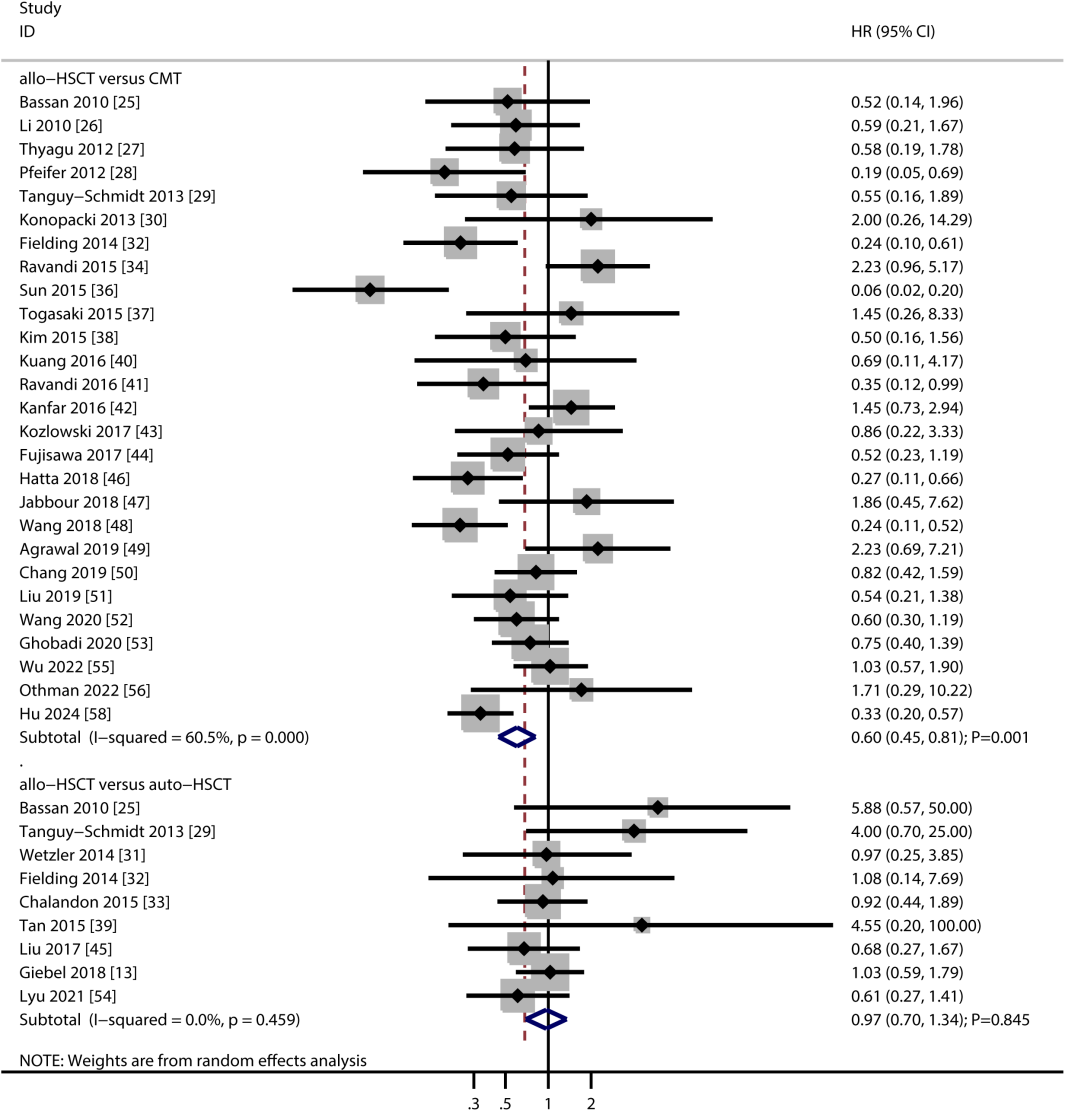
Forest plot summarizing the results for overall survival.

A total of 27 studies compared OS between allo-HSCT and TKI-based chemotherapy, and 9 studies compared allo-HSCT with auto-HSCT (**Figure 2**). Pooled analysis showed that allo-HSCT was associated with significantly improved OS compared to TKI-based chemotherapy (HR: 0.60; 95% CI: 0.45–0.81; *P* = 0.001). In contrast, no significant difference in OS was observed between allo-HSCT and auto-HSCT (HR: 0.97; 95% CI: 0.70–1.34; *P* = 0.845). Substantial heterogeneity was detected in the allo-HSCT vs. TKI-based chemotherapy comparison (*I^2^* = 60.5; *P* < 0.001), whereas no heterogeneity was observed for allo-HSCT vs. auto-HSCT (*I^2^* = 0.0; *P* = 0.459). Sensitivity analyses confirmed the stability of the pooled OS estimates in both comparisons (**Supplementary Figure S1**). Subgroup analyses indicated that the OS benefit of allo-HSCT over TKI-based chemotherapy was consistent in retrospective studies, studies conducted in Eastern countries, studies using imatinib, and high-quality studies. No significant OS differences were observed between allo-HSCT and auto-HSCT across any subgroups (**Table 2**). No evidence of publication bias was detected for either comparison (allo-HSCT vs. TKI-based chemotherapy: Egger’s *P* = 0.861; Begg’s *P* = 0.428; allo-HSCT vs. auto-HSCT: Egger’s *P* = 0.072; Begg’s *P* = 0.118) (**Supplementary Figure S2**).

**Table 2:** Subgroup analyses for OS, DFS, and relapse.

Outcomes	Factors	Subgroups	HR or OR and 95%CI	*P* value	*I^2^* (%)	*P* value for heterogeneity	*P* value between subgroups
OS (allo-HSCT versus CMT)	Study design	Prospective	0.65 (0.32-1.29)	0.216	63.5	0.012	0.620
		Retrospective	0.59 (0.42-0.83)	0.002	61.3	< 0.001	
	Country	Eastern	0.56 (0.38-0.82)	0.003	67.1	< 0.001	0.313
		Western	0.68 (0.43-1.10)	0.114	50.6	0.022	
	TKI type	Dasatinib	0.90 (0.35-2.31)	0.819	73.6	0.023	0.008
		Imatinib	0.41 (0.26-0.66)	< 0.001	61.5	0.002	
		Imatinib or dasatinib	0.76 (0.37-1.57)	0.457	63.2	0.028	
		Imatinib, dasatinib, or ponatinib	0.88 (0.57-1.36)	0.564	0.0	0.464	
		Nilotinib	0.52 (0.25-1.08)	0.080	0.0	0.919	
		Ponatinib	1.80 (0.59-5.46)	0.299	0.0	0.942	
	Study quality	High	0.61 (0.45-0.84)	0.002	62.6	< 0.001	0.581
		Moderate	0.52 (0.17-1.56)	0.242	50.8	0.131	
OS (allo-HSCT versus auto-HSCT)	Study design	Prospective	1.27 (0.73-2.20)	0.396	0.0	0.418	0.229
		Retrospective	0.83 (0.55-1.25)	0.381	0.0	0.518	
	Country	Eastern	0.69 (0.38-1.26)	0.223	0.0	0.473	0.185
		Western	1.12 (0.76-1.66)	0.574	0.0	0.482	
	Study quality	High	1.00 (0.70-1.45)	0.979	2.5	0.406	0.610
		Moderate	0.96 (0.23-4.06)	0.953	24.8	0.249	
DFS (allo-HSCT versus CMT)	Study design	Prospective	0.35 (0.18-0.69)	0.002	61.5	0.024	0.629
		Retrospective	0.42 (0.30-0.58)	< 0.001	68.4	< 0.001	
	Country	Eastern	0.35 (0.24-0.51)	< 0.001	65.4	< 0.001	0.008
		Western	0.50 (0.32-0.79)	0.002	58.2	0.014	
	TKI type	Dasatinib	0.56 (0.28-1.14)	0.112	10.6	0.290	< 0.001
		Imatinib	0.33 (0.21-0.52)	< 0.001	58.7	0.005	
		Imatinib or dasatinib	0.31 (0.12-0.76)	0.011	81.2	0.005	
		Imatinib, dasatinib, nilotinib, or ponatinib	0.52 (0.37-0.73)	< 0.001	–	–	
		Imatinib, dasatinib, or ponatinib	0.71 (0.49-1.04)	0.076	0.0	0.574	
		Nilotinib	0.30 (0.14-0.60)	0.001	0.0	0.516	
		Ponatinib	1.59 (0.28-8.94)	0.597	–	–	
	Study quality	High	0.39 (0.29-0.53)	< 0.001	67.2	< 0.001	0.781
		Moderate	0.59 (0.11-3.16)	0.536	63.5	0.098	
DFS (allo-HSCT versus auto-HSCT)	Study design	Prospective	0.97 (0.56-1.67)	0.904	0.0	0.692	0.831
		Retrospective	0.90 (0.61-1.32)	0.585	0.0	0.748	
	Country	Eastern	1.02 (0.57-1.83)	0.936	0.0	0.626	0.669
		Western	0.88 (0.61-1.28)	0.505	0.0	0.785	
	Study quality	High	0.89 (0.63-1.24)	0.479	0.0	0.874	0.519
		Moderate	1.22 (0.49-3.04)	0.669	–	–	
Relapse (allo-HSCT versus CMT)	Study design	Prospective	0.63 (0.13-2.99)	0.556	–	–	0.456
		Retrospective	0.26 (0.14-0.50)	< 0.001	70.6	< 0.001	
	Country	Eastern	0.15 (0.06-0.36)	< 0.001	64.3	0.016	0.003
		Western	0.54 (0.28-1.05)	0.070	51.5	0.083	
	TKI type	Imatinib	0.16 (0.06-0.40)	< 0.001	58.5	0.034	0.008
		Imatinib or dasatinib	0.53 (0.07-3.83)	0.532	72.8	0.025	
		Imatinib, dasatinib, nilotinib, or ponatinib	0.39 (0.27-0.58)	< 0.001	–	–	
		Imatinib, dasatinib, or ponatinib	0.68 (0.33-1.39)	0.290	–	–	
	Study quality	High	0.27 (0.14-0.52)	< 0.001	70.9	< 0.001	0.676
		Moderate	0.44 (0.15-1.29)	0.133	–	–	
Relapse (allo-HSCT versus auto-HSCT)	Study design	Prospective	0.36 (0.19-0.67)	0.001	0.0	0.724	0.772
		Retrospective	0.40 (0.27-0.59)	< 0.001	0.0	0.820	
	Country	Eastern	0.46 (0.25-0.86)	0.015	0.0	0.964	0.489
		Western	0.36 (0.24-0.53)	< 0.001	0.0	0.886	
	Study quality	High	0.38 (0.26-0.54)	< 0.001	0.0	0.909	0.726
		Moderate	0.45 (0.17-1.22)	0.118	–	–	

### 3.4 Disease-free survival

**Figure 3:**
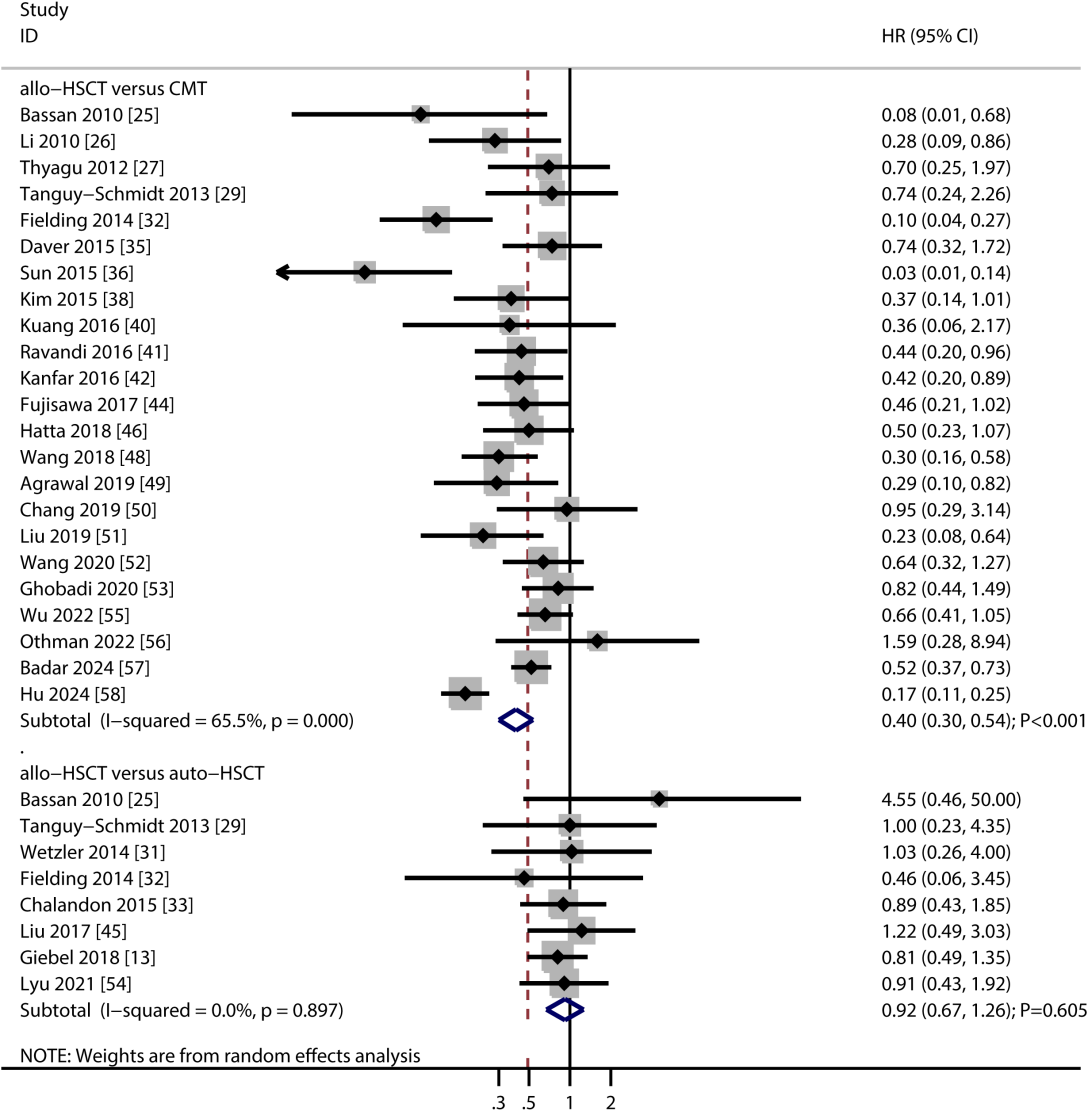
Forest plot summarizing the results for disease-free survival.

Twenty-three studies reported DFS comparisons between allo-HSCT and TKI-based chemotherapy, and eight studies compared allo-HSCT with auto-HSCT (**Figure 3**). Allo-HSCT was associated with significantly better DFS than TKI-based chemotherapy (HR: 0.40; 95% CI: 0.30–0.54; *P* < 0.001). No significant DFS difference was found between allo-HSCT and auto-HSCT (HR: 0.92; 95% CI: 0.67–1.26; *P* = 0.605). Significant heterogeneity was present in the allo-HSCT vs. TKI-based chemotherapy analysis (*I^2^* = 65.5; *P* < 0.001), but not in the allo-HSCT vs. auto-HSCT comparison (*I^2^* = 0.0; *P* = 0.897). Sensitivity analyses supported the robustness of the DFS results (**Supplementary Figure S1**). Subgroup analyses showed that allo-HSCT was associated with improved DFS compared to TKI-based chemotherapy in most subgroups, except in those using dasatinib, imatinib/dasatinib/ponatinib, or ponatinib as TKI therapy, and in studies of moderate quality. No significant DFS differences were observed between allo-HSCT and auto-HSCT in any subgroup (**Table 2**). Publication bias was not detected for DFS outcomes (allo-HSCT vs. TKI-based chemotherapy: Egger’s *P* = 0.654; Begg’s *P* = 0.267; allo-HSCT vs. auto-HSCT: Egger’s *P* = 0.260; Begg’s *P* = 0.266) (**Supplementary Figure S2**).

### 3.5 Relapse

**Figure 4:**
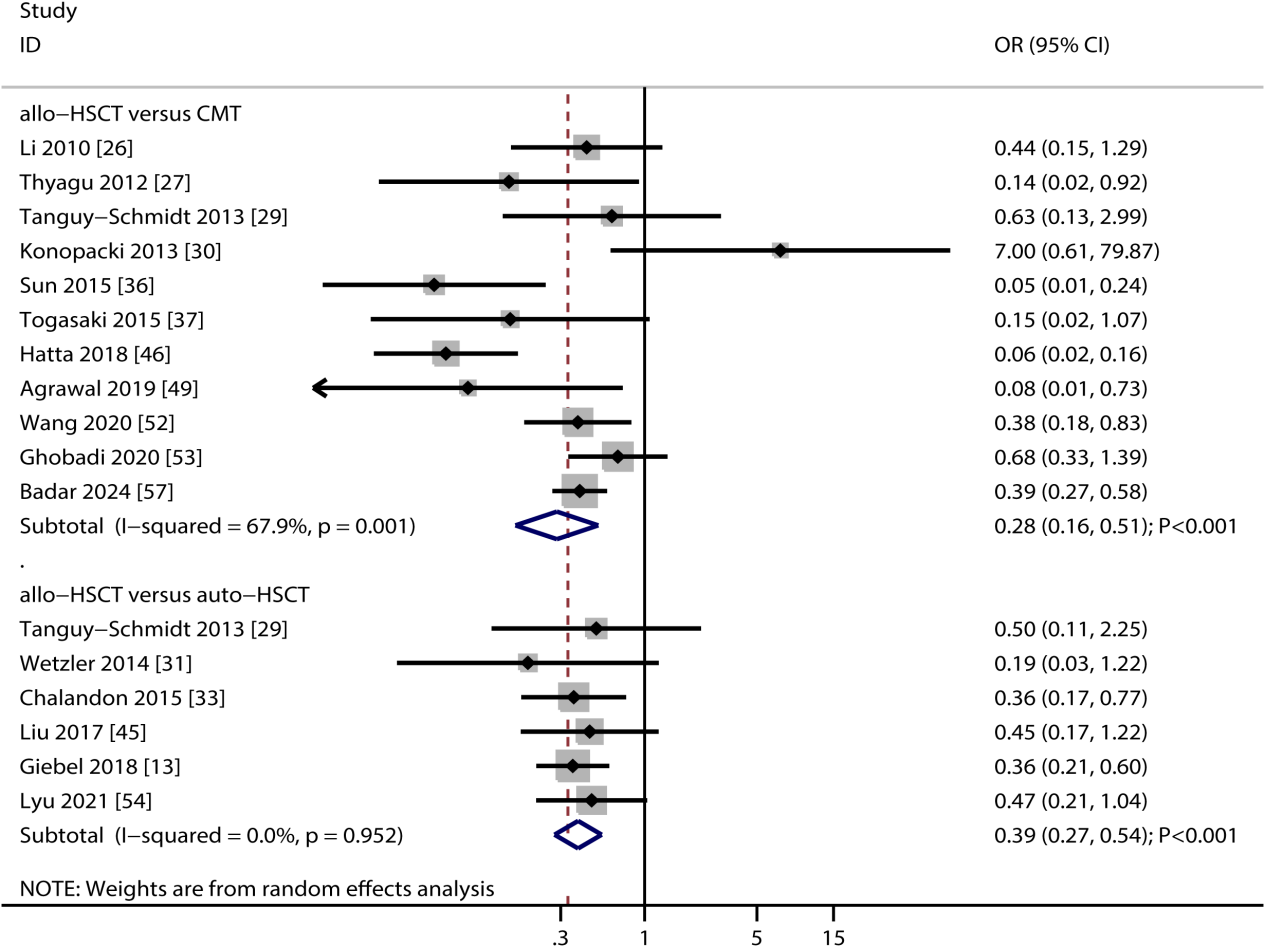
Forest plot summarizing the results for relapse risk.

Eleven studies compared relapse risk between allo-HSCT and TKI-based chemotherapy, and six studies compared allo-HSCT with auto-HSCT (**Figure 4**). Allo-HSCT was associated with a significantly lower relapse risk compared to both TKI-based chemotherapy (OR: 0.28; 95% CI: 0.16–0.51; *P* < 0.001) and auto-HSCT (OR: 0.39; 95% CI: 0.27–0.54; *P* < 0.001). Heterogeneity was substantial in the allo-HSCT vs. TKI-based chemotherapy comparison (*I*² = 67.9; *P* = 0.001) but absent in the allo-HSCT vs. auto-HSCT (*I^2^* = 0.0%; *P* = 0.952). Sensitivity analyses confirmed the stability of these results (**Supplementary Figure S1**). Subgroup analyses indicated that allo-HSCT was associated with a lower relapse risk compared to TKI-based chemotherapy in retrospective studies, studies from Eastern countries, those using imatinib, or mixed TKIs (imatinib/dasatinib/nilotinib/ponatinib), and high-quality studies. A consistent reduction in relapse risk was also observed for allo-HSCT over auto-HSCT across nearly all subgroups, except in studies of moderate quality (**Table 2**). No significant publication bias was detected for relapse outcomes (allo-HSCT vs. TKI-based chemotherapy: Egger’s *P* = 0.431; Begg’s *P* = 0.640; allo-HSCT vs. auto-HSCT: Egger’s *P* = 0.924; Begg’s *P* = 1.000) (**Supplementary Figure S2**).

## 4 Discussion

Our meta-analysis, which included 35 studies involving 3,827 patients with Ph+ ALL, offers comprehensive evidence supporting the therapeutic advantages of allo-HSCT. The key findings indicate that allo-HSCT significantly improves both OS and DFS compared to TKI-based chemotherapy, while yielding survival outcomes comparable to those of auto-HSCT. Importantly, allo-HSCT was associated with a significantly lower risk of relapse than both TKI-based chemotherapy and auto-HSCT, with a particularly pronounced risk reduction observed in comparison with TKI-based regimens. These results remained consistent across multiple sensitivity and subgroup analyses, underscoring the robustness of our conclusions.

Previous meta-analyses have also evaluated the efficacy of allo-HSCT in patients with Ph+ ALL. Ponvilawan et al. (59), who included 26 studies, reported that HSCT led to superior outcomes compared with chemotherapy alone. They also found that auto-HSCT provided survival outcomes similar to those of allo-HSCT in patients without suitable donors or when haploidentical transplantation was not feasible. Similarly, Zeng et al. (60), in a meta-analysis of 15 studies, identified TKI-combined chemotherapy as a viable post-remission treatment option for adult Ph+ ALL patients ineligible for allo-HSCT. However, these earlier studies had notable limitations, such as potential omissions in literature coverage and insufficient exploratory analyses comparing auto-HSCT and allo-HSCT. Our study not only updates prior evidence systematically but also provides in-depth comparative and subgroup analyses to address these gaps.

In the treatment of adult patients with Ph+ ALL, comparative analyses have demonstrated superior survival benefits with allo-HSCT over TKI-based regimens. This advantage can be attributed to two primary mechanisms. First, the myeloablative conditioning regimens used in allo-HSCT allow more comprehensive eradication of leukemic cells, including quiescent leukemic stem cells that are often resistant to conventional therapies. Second, donor-derived hematopoietic stem cells reconstitute normal hematopoietic and immune functions, thereby fundamentally correcting the patient’s dysregulated hematopoiesis (61). Following transplantation, donor immune cells mediate graft-versus-leukemia (GVL) effects by targeting residual malignant cells—an immune-mediated antitumor response that effectively eliminates minimal residual disease. This dual mechanism, combining direct cytoreduction and sustained immune surveillance, significantly reduces relapse rates and prolongs survival (62). Notably, TKI maintenance therapy following allo-HSCT has become a standard of care in recent years, with evidence indicating that it further reduces relapse risk by 30–40% in patients with Ph+ ALL (63). This introduces a potential confounding factor: the favorable outcomes associated with allo-HSCT in our meta-analysis may reflect not only the GVL effect but also the impact of post-transplant TKI maintenance, rather than allo-HSCT alone. Unfortunately, due to limited reporting, we were unable to disentangle this confounding effect. Similarly, among the TKI-based chemotherapy studies, 24 out of 35 (68%) did not provide details on maintenance duration, precluding a fair comparison between “induction plus maintenance” and “allo-HSCT with or without maintenance.”

Notably, allo-HSCT can overcome TKI resistance, which develops in approximately 30–40% of patients receiving long-term TKI therapy. Even in TKI-resistance cases, allo-HSCT exerts antileukemic effects through alternative mechanisms—such as GVL cytotoxicity and the elimination of chemotherapy-insensitive cells—thereby offering a viable salvage treatment option. The substantial heterogeneity observed in comparisons between allo-HSCT and TKI-based therapy can be largely explained by the type of TKI used. For first-generation imatinib, allo-HSCT provides clear benefits in both OS and DFS, as imatinib’s limited efficacy against resistant clones (e.g., T315I mutation) increases reliance on the GVL effect conferred by allo-HSCT. In contrast, second-generation dasatinib and third-generation ponatinib—with their broader mutation coverage and deeper MRD)clearance—attenuate this benefit, to the extent that allo-HSCT no longer significantly improves OS or DFS compared to these potent TKIs. This observation aligns with the notion that “not all TKIs are equal” and helps explain why earlier studies demonstrated a stronger superiority of allo-HSCT, whereas more recent trials have questioned its necessity in the era of advanced TKIs. Subgroup analyses further revealed that the survival advantage of allo-HSCT over TKI-based therapy was more pronounced in retrospective studies and Eastern populations, possibly reflecting regional variations in TKI accessibility and transplant expertise. The absence of significant heterogeneity in comparisons between allo-HSCT and auto-HSCT suggests greater standardization of clinical practices within transplantation settings.

The superior relapse control achieved with allo-HSCT may be attributed to a dual mechanism: the synergistic interaction between the GVL effect and the intensified conditioning regimen (64). The GVL effect represents the core immunological advantage of allo-HSCT, wherein immune cells—such as donor-derived T cells and natural killer cells—recognize and target leukemia-specific antigens as non-self. The intensified conditioning regimen establishes a foundation for this response: high-dose chemotherapy, often combined with radiotherapy, effectively eliminates leukemia cells—including drug-resistant and quiescent leukemia stem cells—thereby reducing tumor burden. This not only facilitates the engraftment of donor hematopoietic stem cells but also minimizes the resistance of residual leukemia cells to the GVL effect. This immunological benefit is particularly critical in Ph+ ALL, where persistent MRD following chemotherapy remains a major clinical challenge. Notably, despite the superior relapse control observed with allo-HSCT, no statistically significant difference in OS was detected between allo-HSCT and auto-HSCT. This finding merits further investigation. Potential explanations include transplantation-related mortality offsetting the survival benefit from relapse reduction, or differences in the efficacy of salvage therapies following disease recurrence.

A transformative trend in the management of adult Ph+ ALL is the integration of immunotherapies, particularly bispecific T-cell engagers such as blinatumomab (anti-CD19/CD3). In contrast to intensive chemotherapy or transplantation, blinatumomab targets malignant B-cell precursors with high specificity, thereby reducing off-target toxicity and treatment-related mortality—key limitations of conventional regimens (65, 66). Notably, MD Anderson Cancer Center has pioneered efforts to optimize transplant utilization in the immunotherapy era, proposing a “transplant-deferral” strategy in which frontline therapy combines blinatumomab with TKIs to achieve deep and sustained molecular remission, while reserving allo-HSCT exclusively for patients with disease progression, persistent MRD, or TKI resistance (67). Our findings should be interpreted within this evolving paradigm: although allo-HSCT remains superior to TKI-based chemotherapy in preventing relapse, blinatumomab may narrow this gap by enabling improved long-term disease control with reduced toxicity. For patients ineligible for allo-HSCT, blinatumomab may represent a viable alternative to auto-HSCT, although long-term data on relapse rates are still limited. Future meta-analyses should incorporate direct comparative data between blinatumomab-containing regimens and HSCT to further refine treatment algorithms.

An important consideration for patients ineligible for allo-HSCT is whether auto-HSCT combined with TKI maintenance offers advantages over second- or third-generation TKI monotherapy. Currently, direct comparative evidence is lacking, as no randomized trials or large cohort studies have addressed this question. However, indirect data can inform clinical decision-making. For patients with access to second- or third-generation TKIs, monotherapy maintenance is generally preferred (67), as it yields 3-year leukemia-free survival rates comparable to those of auto-HSCT plus TKI, while avoiding transplant-related risks. Auto-HSCT with TKI maintenance remains a valuable option in two scenarios: (1) low- and middle-income countries or resource-limited settings where advanced-generation TKIs are unavailable; and (2) patients with persistent MRD positivity despite first- or second-generation TKI therapy. Future head-to-head trials are warranted to clarify the optimal strategy in this population.

Several limitations of this study should be acknowledged. First, the predominance of retrospective studies (24/35) introduces potential selection bias. Second, geographic imbalance may limit the generalizability of our findings, particularly given regional disparities in healthcare resource allocation. Third, substantial heterogeneity in TKI-based chemotherapy and HSCT protocols could not be fully explored due to insufficient reporting of relevant data, which limits our ability to identify subgroups that may derive greater benefit from specific therapies. Fourth, variability in TKI treatment duration and transplant conditioning regimens precludes precise protocol-specific recommendations. Fifth, we were unable to account for the role of TKI maintenance therapy—now a standard of care following both allo-HSCT and chemotherapy—which represents a critical unmeasured confounder. Finally, this meta-analysis shares the inherent limitations of all studies based on published literature, including potential publication bias and restricted granularity for subgroup analyses.

## 5 Conclusions

This comprehensive meta-analysis establishes allo-HSCT as the optimal consolidative therapy for Ph+ ALL, demonstrating superior survival outcomes and relapse control compared to TKI-based regimens. Importantly, while allo-HSCT and auto-HSCT showed comparable survival benefits, allo-HSCT maintained superior antileukemic efficacy, reducing the risk of relapse by 61% compared to auto-HSCT. Based on the synthesized evidence, we propose the following therapeutic algorithm: (1) Allo-HSCT for eligible patients with suitable donors should be prioritized for patients receiving first-generation imatinib, those exhibiting early TKI resistance, or those with high-risk genetic features. For patients on second- or third-generation TKIs (e.g., dasatinib, ponatinib) who achieve durable MRD negativity, allo-HSCT may be deferred in favor of continued TKI maintenance; (2) Auto-HSCT combined with at least 12 months of post-transplant TKI maintenance may be considered for a select group of patients ineligible for allo-HSCT, specifically those who sustain durable complete molecular remission on TKI therapy, lack access to second- or third-generation TKI monotherapy, or have a history of persistent MRD positivity. It is important to note that when available, second- or third-generation TKI monotherapy is generally preferred over auto-HSCT plus TKI, as current evidence does not demonstrate the superiority of the auto-HSCT strategy; and (3) In regions with limited transplant access or for patients unable to tolerate transplantation, prolonged TKI/chemotherapy combinations or blinatumomab-based immunotherapy may serve as a bridge to delayed transplantation or as long-term maintenance for patients who achieve MRD negativity.

## Data Availability

The original contributions presented in the study are included in the article/**Supplementary Material**. Further inquiries can be directed to the corresponding author.
