# Supplementary material for: Comparing therapeutic effects of hematopoietic stem cell transplantation, tyrosine kinase inhibitors and chemotherapy in adult patients with Philadelphia chromosome-positive acute lymphoblastic leukemia: a systematic review and meta-analysis
Source: Front Oncol. 2025 Oct 15;15:1627825. doi: 10.3389/fonc.2025.1627825 (PMC12568404; doi:10.3389/fonc.2025.1627825)
Supplement: Supplementary file 1 [file DataSheet1.docx]

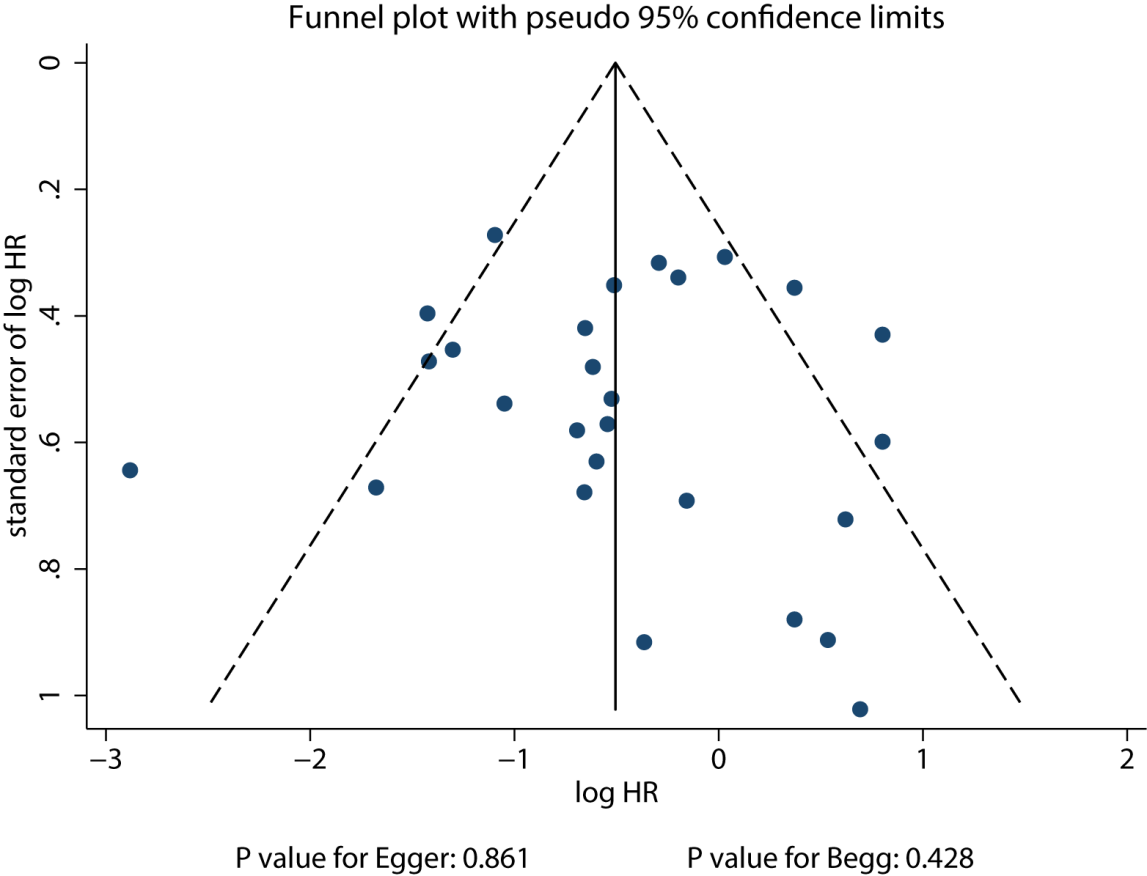


Figure S1. Funnel plot for allo-HSCT versus CMT on OS


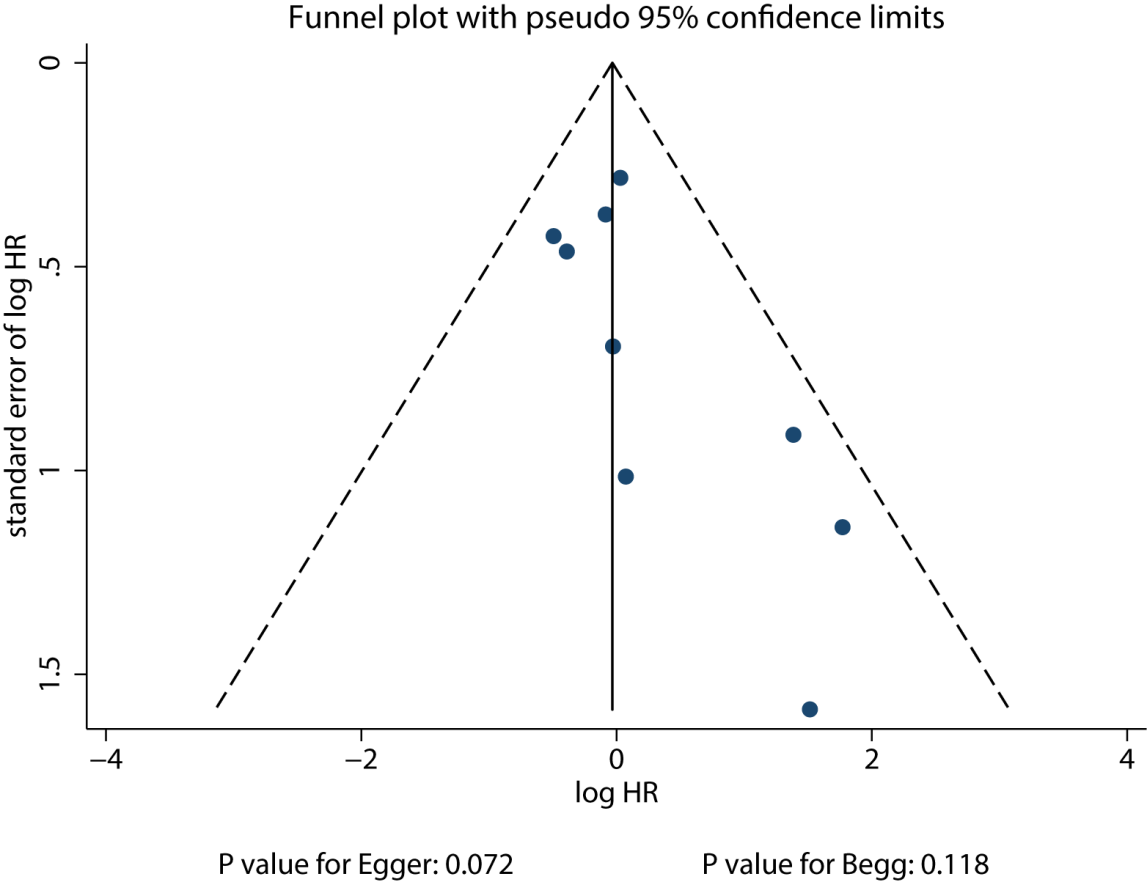


Figure S2. Funnel plot for allo-HSCT versus auto-HSCT on OS


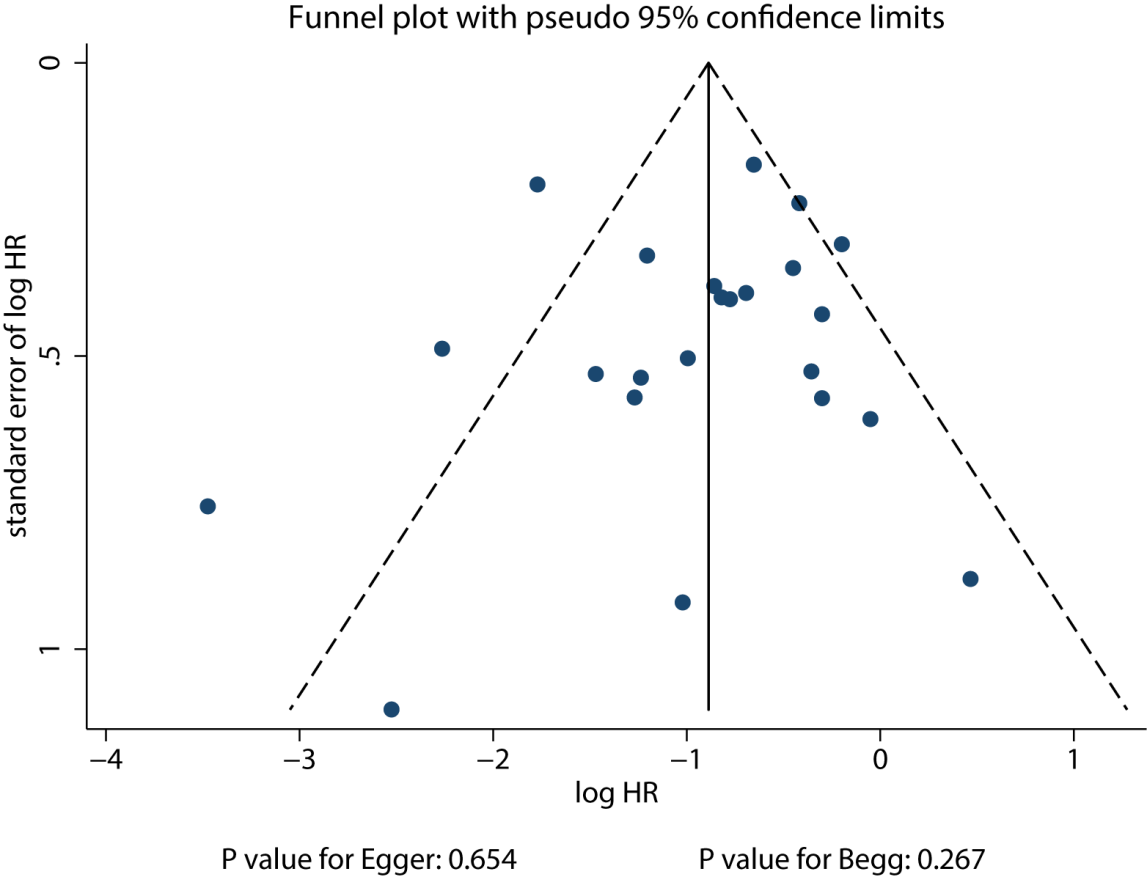


Figure S3. Funnel plot for allo-HSCT versus CMT on DFS


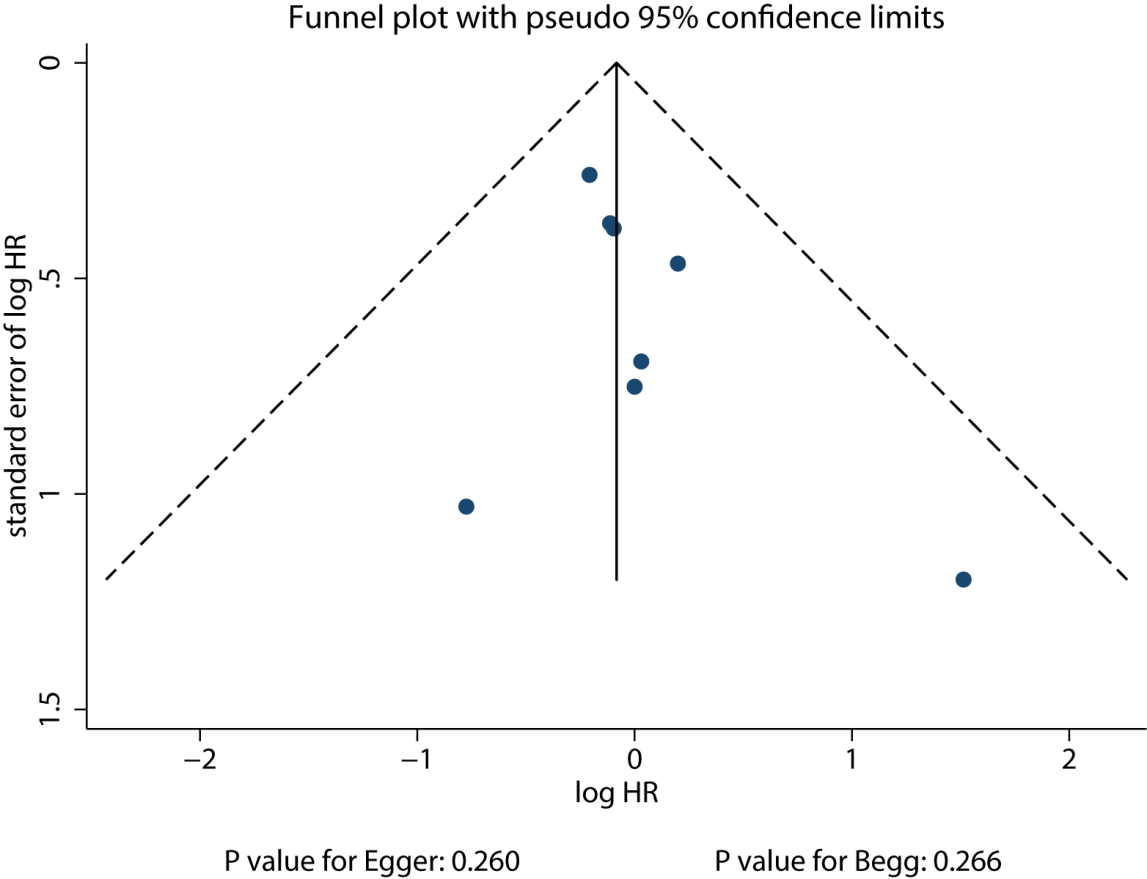


Figure S4. Funnel plot for allo-HSCT versus auto-HSCT on DFS


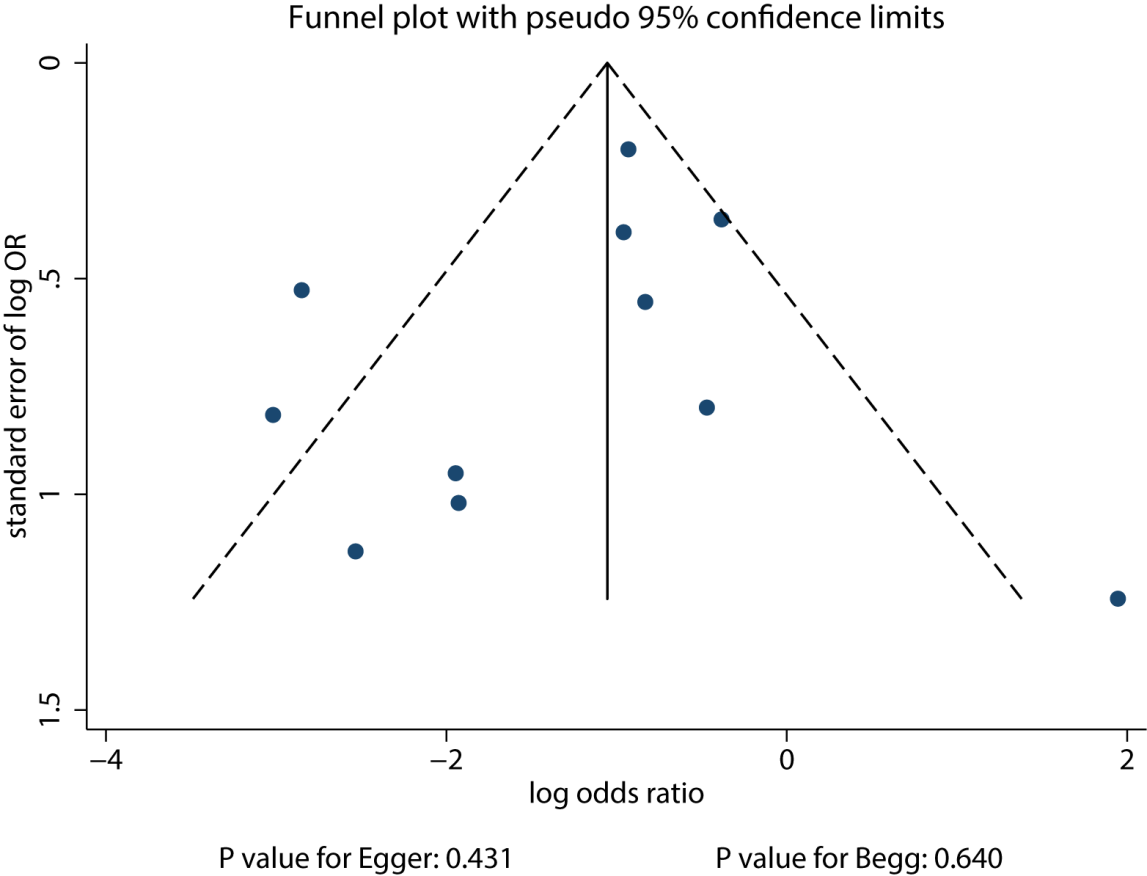


Figure S5. Funnel plot for allo-HSCT versus CMT on the risk of relapse


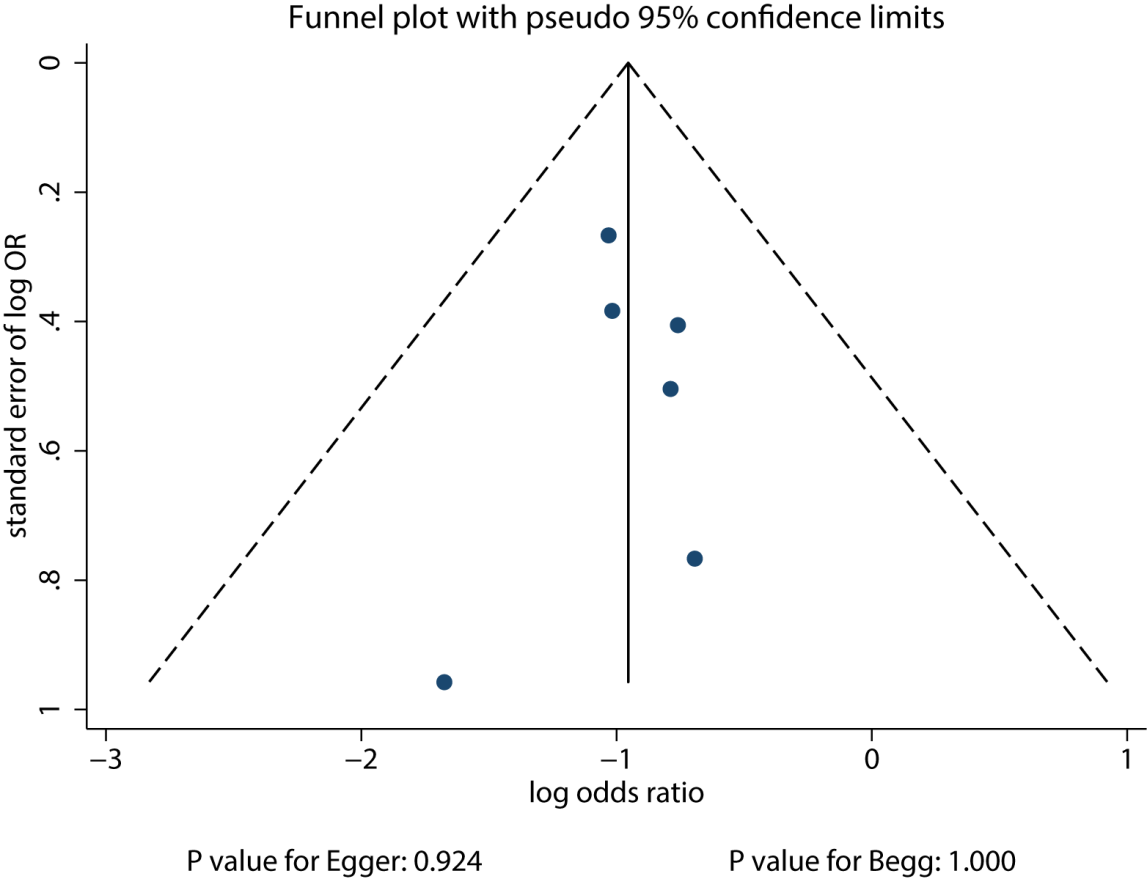


Figure S6. Funnel plot for allo-HSCT versus auto-HSCT on the risk of relapse
